# Links between Childhood Obesity, High-Fat Diet, and Central Precocious Puberty

**DOI:** 10.3390/children10020241

**Published:** 2023-01-29

**Authors:** Valeria Calcaterra, Vittoria Carlotta Magenes, Chiara Hruby, Francesca Siccardo, Alessandra Mari, Erika Cordaro, Valentina Fabiano, Gianvincenzo Zuccotti

**Affiliations:** 1 Department of Internal Medicine, University of Pavia, 27100 Pavia, Italy; 2 Pediatric Department, Buzzi Children’s Hospital, 20154 Milano, Italy; 3 Department of Biomedical and Clinical Science “L. Sacco”, University of Milano, 20157 Milano, Italy

**Keywords:** childhood obesity, high-fat diet, central precocious puberty, pediatrics, children

## Abstract

In recent years, the existing relationship between excess overweight and central precocious puberty (CPP) has been reported, especially in girls. Different nutritional choices have been associated with different patterns of puberty. In particular, the involvement of altered biochemical and neuroendocrine pathways and a proinflammatory status has been described in connection with a high-fat diet (HFD). In this narrative review, we present an overview on the relationship between obesity and precocious pubertal development, focusing on the role of HFDs as a contributor to activating the hypothalamus–pituitary–gonadal axis. Although evidence is scarce and studies limited, especially in the paediatric field, the harm of HFDs on PP is a relevant problem that cannot be ignored. Increased knowledge about HFD effects will be useful in developing strategies preventing precocious puberty in children with obesity. Promoting HFD-avoiding behavior may be useful in preserving children’s physiological development and protecting reproductive health. Controlling HFDs may represent a target for policy action to improve global health.

## 1. Introduction

Childhood obesity is one of the most severe health challenges of the 21st century [1]. Obesity has a profound impact on children and adolescents. Firstly, children with obesity are exposed to related complications such as metabolic alterations, cardiovascular diseases, orthopedic complications, and psychosocial disorders; secondly, affected children have a five-fold increased risk of staying obese as adults, influencing adult longevity [2,3,4,5,6].

The pathogenic mechanism of obesity is multifactorial; however, the main cause of childhood obesity is a positive energy balance due to increased caloric intake and reduced energy expenditure, typical of sedentary lifestyles. Since the major cause of these global health issues is potentially reversible, major research efforts have been deployed to analyze factors that lead to a positive energy balance and define potential strategies for children and their families to maintain a healthy lifestyle [7].

In recent years, the existing relationship between excess overweight and central precocious puberty (CPP) has been reported, especially in girls. This assumption is based on the observation that early-onset puberty is often associated with excess body weight and that both conditions’ prevalence has risen in past decades [8]. Even though the mechanisms underlying this association remain elusive, the role of diet has been studied. Different nutritional choices have been associated with different patterns of puberty. In particular, the involvement of altered biochemical and neuroendocrine pathways and a proinflammatory status has been described in connection with a high-fat diet (HFD) [9,10,11,12,13,14].

Several studies highlighted the association between overweight/obesity and PP development. Recent evidence suggests indeed that in children, diets with HF content and excess body weight are factors influencing pubertal timing [15,16,17,18,19,20,21,22]. Although the role played by nutritional status in pubertal activation has gained more and more attention in recent years, studies concerning the process behind this delicate theme are scarce [18,19,20,21]. Recent works propose different mechanisms to explain this association, as later reported in the text. In any case, it is still unclear which of the mechanisms described have a major role in the development of PP in obese children [17,18,19]. The majority of studies focus their attention on the role of high ectopic fat deposition, which seems to promote hypothalamic inflammation, altering the GnRH network both via phoenixin action and via hypothalamic microglial cell activation [18,23,24,25]. According to this theory, an unbalanced diet rich in fats promotes obesity and then PP activation in a weight-dependent manner [17,18,19]. Not all authors agree on this issue, and PP activation has also been reported through mechanisms independent of weight status; the cause–effect relationship remains not fully elucidated.

In this narrative review, we present an overview on the relationship between obesity and precocious pubertal development, focusing on the role of HFDs as a contributor to activating the hypothalamus–pituitary–gonadal axis. Increased knowledge about HFD effects will be useful in developing strategies preventing precocious puberty in children with obesity.

## 2. Methods

We performed a narrative review, summarizing the literature to answer our research question on the role of HFD in the development of precocious puberty in children with obesity. Relevant English-language literature in the past 15 years, including original papers, meta-analyses, clinical trials, and reviews, were independently reviewed and included by the authors V.C.M., F.S., C.H., A.M., and E.C. Case reports or series and letters were not considered. PubMed, Scopus, EMBASE, and Web of Science were adopted as electronic databases for the research. The following keywords (alone and/or in combination) were used for the research: precocious puberty, early puberty, timing of puberty, nutrition, diet, obesity, excess weight. The contributions were collected by V.C., V.C.M., F.S., C.H., A.M., E.C., and V.F. and critically reviewed by V.C., V.F., and G.Z. The resulting draft was discussed with all co-authors, and the final version was approved by all.

## 3. Obesity and Precocious Puberty

### 3.1. Childhood Obesity

Obesity is characterized by increased adipose tissue (AT) mass, leading to severe health problems. It is often defined by the body mass index (BMI), measured by dividing the body weight in kilograms by height in meters squared (kg/m^2^). According to the European Society for Paediatric Endocrinology, children and adolescents > 2 years of age are defined as overweight if the BMI is >85th percentile but <95th percentile and obese if the BMI is ≥95th percentile for age and gender on the CDC charts. Children < 2 years of age are diagnosed as obese if the weight/length ratio is ≥97th percentile of the WHO growth standards [26].

Worryingly, estimates show that about 38.5 million children aged <5 years and more than 340 million children aged 5–19 were overweight or obese in 2019, indicating a dramatic increase in the prevalence of excess body weight in the last four decades [1,27]. Furthermore, although once considered high-income country problems, overweight and obesity are now on the rise in low- and middle-income countries, especially in urban settings. In Africa, the number of overweight children has increased by 25% in the last two decades, and almost half of the overweight children live in Asia [1].

Researchers acknowledge that the main driver of the current overweight epidemic is excess energy intake, mainly due to the expanding ‘obesogenic’ environment, influencing children’s propensity to consume energy-dense foods and drinks and promoting sedentary lifestyles [28]. Nevertheless, obesity results from a complex interplay between environmental, behavioral, biological, and genetic factors [29]. Genome-wide association studies have reported the connection between multiple gene variants and the predisposition to weight gain in an obesogenic environment [30,31]. In addition, single-gene mutations, such as abnormalities of the leptin pathways or the melanocortin-4 receptor, are responsible for early-onset severe obesity associated with hyperphagia, physical dysmorphisms, and other possible endocrine dysfunctions [32,33]. Rising interest in the interaction between genes and the environment has also pointed out the role of epigenetics in obesity pathogenesis. Intrauterine life and early life events can affect gene expression and activity mostly by DNA methylation or histone modification, causing an inheritable risk of weight gain. Factors such as maternal and paternal dietary habits and weight status, maternal smoking during pregnancy, birth weight, breastfeeding, and exposure to air pollutants are mostly examined [34,35,36,37,38,39,40]. However, genetic obesities account for less than 1%. Additionally, rare syndromic and endocrine disorders such as the Prader–Willi, Bardet–Biedl, Alström, and WAGR syndromes, as well as growth hormone (GH) deficiency, primary hypothyroidism, cortisol excess, polycystic ovarian syndrome, and pseudohypoparathyroidism, can present with obesity, usually associated with additional signs and symptoms.

With the increasing prevalence of pediatric obesity, complications usually seen in adults have also been identified in the pediatric age. Obesity is a proinflammatory state that affects nearly every organ and leads to severe comorbidities encompassing, but not limited to, hypertension, dyslipidemia, insulin resistance, diabetes, fatty liver disease, cardiovascular disease, asthma, sleep apnea, and osteoarthritis [41].

In addition, psychological complications associated with pediatric obesity include low self-esteem, depression, body dysmorphic disorder, impaired social relationships, bullying, social stigma, and decreased health-related quality of life.

Lifestyle interventions are feasible and effective in preventing and treating childhood obesity. Medical treatment and/or bariatric surgery are available for a selected pediatric population who fail to achieve their weight loss goals with lifestyle modification therapy alone [42].

### 3.2. Precocious Puberty

Puberty activates the hypothalamus–pituitary–gonadal (HPG) axis, leading to psychological and physical maturation, accelerated linear growth, development of secondary sexual characteristics, and gonadal maturation with the acquisition of reproductive capacity [43]. The first sign of pubertal activation is thelarche in females and testicular enlargement in males, followed by accelerated linear growth and development of secondary sexual characteristics. Menarche usually follows after 2.5 years (median age 12.5 years). Clinical assessment of puberty is usually made according to the Tanner stages of puberty [44,45].

Precocious puberty (PP) is defined as sexual development in females and males before 8 and 9 years of age [44], respectively. The anticipation of menarche was described from the early 19th to mid-20th centuries due to significant improvements in economic and hygienic conditions. This trend has slowed in recent decades [46]. Few studies, however, could demonstrate a marked decline in thelarche age in the last 20 years, raising concern among the scientific community about whether to lower the age limit of evaluation for precocious puberty. Puberty anticipation in males has also been described in recent years, but with a less marked trend [47,48].

PP is categorized in central and peripheral puberty based on the underlying pathogenetic mechanism. Central precocious puberty (CPP) is caused by activation of the hypothalamus–pituitary–gonadal axis and is, therefore, gonadotropin-dependent. This form of PP is the most common, accounting for 80% of cases. In the vast majority of patients, the cause is idiopathic, but patients should undergo a full evaluation for secondary PP with particular attention to neurological signs suggesting an underlying brain tumor. Peripheral precocious puberty (PPP) is far less common and is caused by an increase in sex hormone levels (estradiol and testosterone) from exogenous and endogenous sources [49].

The gold standard for differential diagnosis between the two conditions is the GnRH stimulation test, which evaluates the LH peak and LH-to-FSH ratio after the stimulus. Elevated LH basal levels can diagnose CPP without stimulation tests, with a proposed cut-off of 0.2 mUI/L. However, a stimulation test is still required in patients with lower basal LH levels and clinical signs of pubertal maturation [50]. In recent years, there has been an effort to develop less invasive and time-consuming diagnostic tests, such as measuring urinary LH; however, further research is needed to better assess the diagnostic accuracy of these new methods [51]. Measuring estradiol, testosterone, and adrenal androgens may be useful [52]. Diagnostic evaluation of patients with PP also involves bone age assessment via non-dominant hand radiography due to skeletal maturation induced by sex hormones. A skeletal age of two or more SD compared to chronological age is considered pathological [53]. Initial evaluation with pelvic ultrasound to identify patients with ongoing gonadal maturation (i.e., uterus and ovary enlargement) is also recommended and may require further examination with a GnRH stimulation test [53]. High levels of estradiol and testosterone with basal or stimulated LH or LH/FSH below the cut-off in children with signs of pubertal activation strongly suggest PPP. Tumoral marker evaluation may also be needed to exclude an underlying oncological disease [43,52].

Management of CPP is based on the regular administration of GnRH analogues, which avoid pulsatile secretion by the CNS, as usually occurs in puberty. There is no consensus amongst the scientific community on the timing of treatment suspension, although it may be considered at a bone age of 12–12.5 years for females and 13–13.5 years for males [54]. PPP treatment depends on the underlying etiology [52].

### 3.3. Precocious Puberty in Children with Obesity

Several studies have highlighted the association between overweight and obesity and early breast development and menarche in girls. The situation is less defined in boys: overweight boys mature earlier, whereas obese boys mature later [8,20,55].

A recent cross-sectional study evaluated PP prevalence among Chinese children with different BMIs. The analysis showed a positive correlation between pubertal signs and BMI in both sexes, with a more marked correlation in girls despite the male population having higher BMIs. The median age at Tanner stage 2 was significantly higher for girls with lower BMIs, suggesting that severe thinness impacts sexual development [55]. In a large longitudinal cohort study published by Li et al., BMI, waist circumference (WC), and body composition indicators were measured by bioelectrical impedance and related to early puberty onset in both boys and girls. The analyses noted that the higher the level of adiposity accumulation, the greater the risk of earlier puberty in both sexes, except for smaller magnitudes in boys. Furthermore, body composition, rather than BMI and WC, had a greater impact on the risk of earlier puberty onset. Considering body fat distribution rather than BMI may be relevant since the adipokine profile depends on body fat distribution, and BMI can be falsely elevated in children with a high degree of muscle mass. We also considered the trajectory of anthropometric parameters. Aligning with previous studies, increasing trajectories of fat accumulation increased the risk of earlier puberty onset in both boys and girls [56].

Contrasting the reported evidence, a longitudinal study by Zhai et al. and some cross-sectional surveys suggested that excessive weight at prepubertal age may delay sexual development in boys [51]. Further research is needed to better understand the relationship between puberty and obesity in males.

The hypothesis that a target BMI is needed to initiate puberty was developed in the early 1970s by Frisch et al. Nowadays, it is not considered plausible [57]. Researchers have been trying to identify the underlying mechanisms of adipocytes on pubertal activation, and the most plausible theories are based on the endocrine effect of fat tissue, investigating adipokine secretion, insulin resistance, and peripheral aromatase action [58]. White adipose cells can act as an endocrine organ secreting adipokines that regulate primary feeding behavior. The plasticity of adipocytes and their interaction with the central nervous system is known as the ‘brain–adipose axis’ [59]. Surprisingly, several studies have shown a neurotrophic and neuroprotective role of fat tissue despite mainly regulating appetite and energy metabolism [51]. Regarding the context of reproductive health, multiple metabolic signals regulate the HPG axis. The leptin–kisspeptin interplay is the most studied. The appetite-inhibiting hormone leptin acts as a long-term regulator of body mass level, depressing the activity of the so-called “neurons of hunger” [60]. By binding to the leptin’s receptor, it activates the kisspeptin’s pathway, which communicates to the hypothalamic GnRH pulse secretor that there are sufficient energy stores for fertility to start. This process is necessary but not part of the sufficient criteria for sexual development [61,62]. It has been demonstrated that leptin concentrations rise before puberty onset in girls, and leptin concentration peaks precede the gonadotropin ones [63,64]. Additionally, a negative correlation between leptin concentration and menarche age has been found in girls [65]. Conversely, these correlations have not been demonstrated in boys [63]. Hypothalamic expression of kisspeptin is known to be sexually dimorphic in humans: the hypothalamus of females is programmed to express significantly higher kisspeptin levels than their male counterparts [66]. Subsequently, differences between boys and girls may be referred to as a differential hypothalamic control already present before puberty. Another metabolic signal is ghrelin, which activates when the stomach is empty and has a negative energy balance. In the arcuate nucleus of the hypothalamus (ARC), it suppresses kisspeptin expression. Additionally, receptors of other adipokines, such as adiponectin and resistin, are expressed in the hypothalamus, pituitary gland, and gonads. Adiponectin inhibits GnRH neurons by AMP kinase pathways [67]. Lower circulating concentrations of adiponectin found in children with excess body weight compared to those with a healthy weight may be an additional factor in advancing the onset of puberty [68]. Adipokines have also been associated with insulin resistance, which is one of the hallmarks of obesity. A linear relationship between insulin secretion and leptin expression has also been observed. Adiponectin, reduced in obese children, has an insulin-sensitizing activity [58]. Hyperinsulinemia results in reduced concentrations of sex-hormone-binding globulin, increasing sex steroids’ bioavailability in overweight children and potentially changing the timing of puberty [69]. Finally, the role of adipose tissue in reproductive health is executed centrally and peripherally: adipose cells have an aromatase action converting androgens to estrogens.

**Figure 1:**
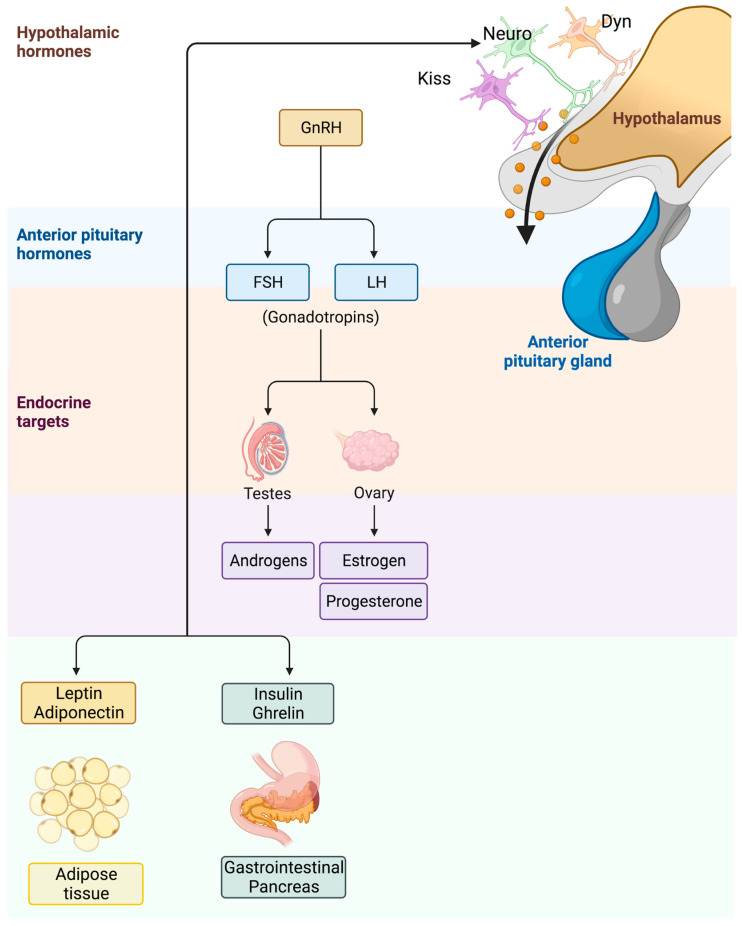
Link between obesity and the activation of the hypothalamic–pituitary–gonadal (HPG) axis. Peripheral signals deregulated in subjects with obesity, such as leptin, adiponectin, insulin, and ghrelin levels, are involved in the regulation of the HPG axis, influencing timing of puberty.

In Figure 1, the link between obesity and PP is schematized.

The evaluation and diagnosis of CPP in overweight patients constitute a challenge for the clinician due to difficulties in clinically assessing puberty and the direct effect of fat tissue on gonadotropin levels and bone maturation.

Mammary gland development stages in females may lead to excess inter-observer variability due to abundant adipose tissue surrounding the glands [55]. Fat may prevent the clinician from palpating a small breast gland in the earliest stages of development and may otherwise cause breasts to look more abundant and lead to over-staging, according to Tanner’s methodology. Although less rapid and economical than clinical evaluation, ultrasound may be needed in patients with obesity [57,70].

Higher BMI, compared to normal-weight patients, impacts LH and FSH secretion through a down-regulation of the HPG axis due to excess circulating estrogen and androgen levels. Lower basal levels of LH in pubertal girls with obesity have been reported in previous decades [71]. Basal and after-stimulus LH levels had inferior results in overweight compared to normal-BMI patients. A more marked reduction has been seen in girls with obesity than in overweight ones [71,72].

Moreover, it has long been acknowledged that body fat induces linear growth and bone maturation even without HPG axis activation [72,73,74]. This finding is a further confounding factor in diagnosing CPP in patients with obesity: bone maturation raises the suspicion of true PP more than the development of secondary sexual characteristics [72]. In obese patients with advanced bone age, predicted adult height may be preserved due to faster linear growth in early childhood, compensating for earlier epiphysis closure during adolescence [75,76]. GnRH therapy appears to be effective in increasing adult height for patients without obesity [72].

## 4. High-Fat Diet in Children with Obesity

### High-Fat Diet and Obesity

The worrisome rise in pediatric obesity reported in the last four decades is concomitant with the profound changes in many countries’ dietary and lifestyle patterns [1,77]. An American study conducted at the end of the last century showed that, in the US, the proportion of calories derived from fats gradually increased from 32 to 43%, whereas calories derived from carbohydrates reduced from 57 to 45% [78].

In European and Mediterranean countries, adults, children, and adolescents adhere less to the Mediterranean diet, and their dietary regimens now resemble Western, high-fat diets [79,80].

In children, eating behaviors and the consequent risk of obesity are associated with different factors, ranging from parental feeding styles and stress to perinatal factors such as birth size and breastfeeding status [81]. Parental obesity is a strong predictor of excess weight gain in children, reflecting a genetic predisposition to weight gain and environmental effects [82]. Moreover, multiple studies conducted in pediatric populations found an association between lack of sleep or inadequate sleeping habits and unhealthy dietary patterns, including HFDs [83,84,85].

An elevated consumption of energy-dense food and HF food has been associated with childhood obesity and increased adiposity [86,87,88,89].

HFD’s impact on obesity and obesity-related complications is not limited to excessive caloric intake. In fact, HFDs have a pivotal role in developing and maintaining obesity due to the complex biochemical and neuroendocrine pathways it triggers.

The connection between unhealthy diets and related diseases has been extensively studied in previous years. Alterations to signalling pathways and the neuroendocrine changes caused by nutritional excess are particularly focused on. Furthermore, a low-grade level of inflammation has been associated with HFDs, among other findings (9–14). Specifically, saturated fatty acids contained in meat, dairy products, and processed foods have proinflammatory effects, whereas unsaturated fatty acids tend to reduce inflammation [89,90]. Metabolic inflammation is defined as the sustained, low-grade activation of different inflammatory molecules, including interleukin, C-reactive protein (CRP), tumor necrosis factor-alpha (TNFα), fibrinogen, and others, registered in over-nutrition-related pathologies such as obesity and type 2 diabetes [91,92,93].

Adipokines are biologically active molecules secreted by adipose tissue, which participate in various homeostatic processes, including energy metabolism, hunger and satiety regulation, insulin sensitivity, and inflammation [94]. A dysregulation of adipose tissues, such as the one induced by a high-fat diet, may impact all these physiological functions through an impaired secretion of adipokines. Research in neuroendocrinology suggests that activated circulating cytokines and adipokines typical of metabolic inflammation are not only a passive effect of incorrect dietary regimens but also play an active role in the development of obesity and obesity-related complications [13,14,95,96,97].

Particular emphasis has been placed on hypothalamic inflammation since energy homeostasis is mainly regulated by the hypothalamus [98,99,100]. Energy homeostasis is physiologically balanced through the melanocortin system, which consists of two functionally antagonistic neuronal populations: Neurons expressing the orexigenic neuropeptides agouti-related peptide (AgRP) and neuropeptide Y (NPY) and neurons expressing the anorexigenic peptides proopiomelanocortin (POMC), cocaine- and amphetamine-regulated transcript (CART) [101,102,103]. Circulating levels of leptin and insulin, which vary according to nutritional status, activate POMC neurons and inhibit AgRP neurons, affecting appetite and energy intake [104].

HFD has been associated with hypothalamic inflammation through different mechanisms. Firstly, high-fat meals induce the production of proinflammatory cytokines such as TNFα, IL-4, and TGFβ in the plasma and intestinal tissue [105,106,107,108,109]. Secondly, HFD promotes microglia and astrocyte proliferation in the hypothalamus, which, if over-represented, may increase the release of inflammatory cytokines and impair leptin’s action on POMC neurons. Interestingly, Fujioka et al. reported the existence of intracellular communications mediated by prostaglandins from microglia to GnRH, linking HFD and obesity to precocious puberty [23,110,111].

In addition, signals from Toll-like receptors (TLRs) activate the inhibitor of κB-kinase-β (IKKβ)/nuclear factor-κB (NF-κB) and other intracellular inflammatory signals in response to stimulation by circulating saturated fatty acids, exacerbating the inflammatory response and associated insulin resistance [112]. HFD and the consequent hypothalamic inflammation ultimately lead to some degree of leptin resistance, a hallmark of obesity [113,114].

HFD-induced inflammation negatively affects multiple physiological functions and often overlaps with HFD-induced dysbiosis. The microbiota is highly affected by dietary changes, and its composition may vary depending on nutritional apports [115]. In fact, intestinal bacteria adjust their metabolism according to their interactions with other microbes and the nutrient supply; therefore, unhealthy dietary changes alter the microbiota and influence metabolic and inflammatory pathways [116]. There is considerable evidence linking HFD, microbiota composition, and inflammation [95,117,118,119].

HFD-induced dysbiosis comprises different gut flora alterations, including an overall reduced bacterial count, increased Firmicutes/Bacteroidetes ratio, and increased gut permeability [117,120,121,122,123]. The latter alteration, also known as a leaky gut syndrome, has a multifactorial etiology, comprising reduced gut-barrier-promoting bacteria, a parallel increase in bacteria with a damaging action to the barrier, altered mucus layer due to the proliferation of mucin-degrading bacteria, and increased bacteria genres, which reduce the expression of tight junction proteins [124,125,126,127,128,129].

A direct consequence of leaky gut syndrome is enhanced translocation into the bloodstream of bacteria lipopolysaccharides (LPSs), which acts as a key factor in triggering the low-grade systemic metabolic inflammation cited above [130]. LPS has a direct inflammatory effect, as it promotes intestinal inflammation and epithelial cell shedding with tight junction impairment and an indirect effect through TLR4 activation, generating a signalling cascade that results in inflammatory cytokine release [121]. However, increased levels of LPSs in the plasma of subjects exposed to HFD are not just a consequence of leaky gut syndrome because HFD itself promotes the production of endogenous LPSs through the proliferation of LPS-producing bacteria [130,131].

## 5. High-Fat Diet and Precocious Puberty 

### 5.1. Epidemiological Evidence

Evidence suggests that diets with HF content, strongly related to obesity, and excess body weight are known factors influencing pubertal timing [15,16,17,18,127].

Chen et al. observed through a cross-sectional study that 13.86% of Shanghai girls with PP were obese [132], and several studies underlined the close correlation between Body Mass Index (BMI) and PP [21,22,24,133,134]. Rosenfield et al. reported that girls with a high BMI reached menarche and pubarche earlier than girls with a lower BMI [135], confirming the crucial role played by nutritional status in pubertal activation.

Although the neuroendocrine mechanism is still unclear, it appears that high ectopic fat deposition promotes hypothalamic inflammation and that inflammatory mediators produced by excess body weight alter the GnRH network [23,24,25].

Curiously, HFD seems to affect the onset of puberty independent of body weight [136].

Careful evaluation of different nutritional choices has led to multiple discoveries. A prospective observational study conducted among US girls demonstrated that those with greater adherence to the Mediterranean diet reached puberty later than girls with lower adherence [137]. High intake of vegetable proteins and fibers correlates with delayed menarche in girls [138,139]. Berkey et al. reported that girls aged 3–5 with a vegetable protein intake of approximately 3 g/day reached menarche about 0.87 years later than their peers with a lower intake [140]. The same result is obtained through phytoestrogens, lignin, and flavanol when introduced with good consumption of fruit and vegetables [141]. Conversely, excessive consumption of animal protein correlates with a younger age at menarche [142,143]. In a cohort study, Jansen et al. reported that girls who consumed red meat equal to or greater than two times per day experienced menarche earlier than those who consumed red meat less than four times per week [143]. Furthermore, the habitual consumption of sugar-sweetened soft drinks is positively associated with the risk of earlier puberty [144], whereas evaluations conducted on dairy products report conflicting results [145,146,147]. High total fat and polyunsaturated fatty acid intake have also been associated with earlier menarche [148,149].

However, it is necessary to specify that diets include combinations of different foods, and none are solely sufficient to influence the onset of puberty significantly.

In Table 1, we summarized the manuscripts analyzed to discuss the epidemiological evidence.

### 5.2. Mechanisms Involved

In previous years, childhood obesity and PP have risen [15]. The two conditions—major challenges in our societies—are interconnected and related to nutritional habits [16]. Different molecular and animal studies have indicated a close relationship between diet and fertility, specifically between HFD and PP activation [17,18,19]. Observatory data demonstrate that unhealthy diet patterns are significantly associated with PP in children [17]. Studies concerning the process behind this delicate theme are scarce, but recent evidence proposes different mechanisms to explain this association.

**Figure 2:**
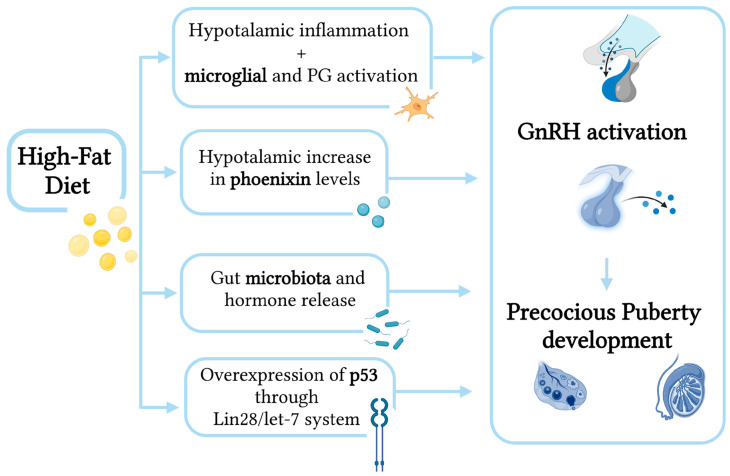
Mechanisms by which a high-fat diet promotes precocious puberty.

In detail (Figure 2), the major mechanisms by which an HFD promotes PP are the following:

1. Activation of GnRH via hypothalamic microglial cells [18];
2. Activation of GnRH via diet-induced phoenixin action [18];
3. Modification of gut microbiota and hormones [19,20];
4. Overexpression of p53 through Lin28/let-7 system [21].

#### 5.2.1. Activation of GnRH via Hypothalamic Microglial Cells

HFD causes hypothalamic inflammation and microglial cell activation. Valdearcos et al. determined that microglia of the hypothalamus are sensitive to fatty acids (FAs) in murine models [22]. The researchers demonstrated that mice’s microglial cells undergo inflammatory activation with a diet rich in saturated FAs [18,24]. Interestingly, FAs (and not obesity per se) induce microglial activation, as shown by Gao et al., who demonstrated that genetically obese mice do not show hypothalamic microglia activation when not fed an HFD [133,134]. Microglial cells play a key role in PP, as these cells communicate with GnRH cells. This intercellular communication was mediated by prostaglandins [23]. GnRH, secreted following a pulsatile pattern from the hypothalamus, leads to the secretion of LH and FSH from the anterior pituitary gland. In conjunction with estradiol and testosterone, these hormones promote oogenesis and spermatogenesis, respectively [23,24]. In addition, microglial cells also enhance the production of brain-derived neurotrophic factors (BDNF) for several neurons, among which are the gonadotrophic axis in the preoptic area and the pituitary gland [25].

#### 5.2.2. Activation of GnRH via Diet-induced Phoenixin Action

The central control of puberty is exercised through GnRH [151]; however, the mechanism by which GnRH varies, according to developmental and metabolic factors, is not clearly understood. The kisspeptin signalling pathway and its activation by phoenixin play a role in the neuronal regulation of GnRH [152]. Phoenixin, a neuropeptide shown to mediate anterior pituitary function in fertility, induces gonadotropin secretion through GnRH stimulation mediated by kisspeptin, which potently stimulates GnRH release [153,154,155]. Kisspeptin’s crucial role in puberty was proven by Seminara et al. and Roux et al., who, through genetic analysis, demonstrated that inactivated mutations of the kisspeptin receptor fail to progress through puberty both in human and animal models [136,156]. Moreover, Nguyen et al. evaluated samples of surgically removed ovarian tissues from eight women of reproductive age (19–32 years old) and demonstrated a direct stimulating effect of phoenixin and its receptor, GPR173, on human ovarian follicles [157]. In the same study, phoenixin was shown to induce estradiol production in a dose-dependent manner [157]. Research has also shown a possible regulation of phoenixin expression by fatty acids [158]. Specifically, McIlwraith et al. showed that fatty acids palmitate, docosahexaenoic acid (DHA), and oleate stimulate phoenixin gene expression. However, the mechanisms of this process have yet to be elucidated [158].

FAs can cross the blood–brain barrier and were shown to regulate hypothalamic peptide expression. In the same study, immortalized hypothalamic neurons were shown to increase phoenixin mRNA levels (measured with RT-qPCR) when treated with palmitate, DHA, and oleate [158]. This result highlights the important concept that dietary signals in the hypothalamus play a role in controlling reproduction [18,158,159].

#### 5.2.3. Modification of Gut Microbiota and Hormones

Since the gut microbiota is associated with hormone secretion and obesity, researchers have focused on the possible relationship between microbiota and PP [19,20,160]. Li et al. evaluated 73 girls (27 girls with central PP, 24 overweight girls, and 22 healthy controls) and investigated their gut microbiota characteristics through 16S rRNA sequencing on fecal samples [20]. The researchers identified several PP-associated bacteria and emphasized the importance of bacterial-synthesized neurotransmitters (such as acetate, dopamine synthesis, and nitric oxide) on PP [20]. Moreover, many studies have highlighted how gut microorganisms connect and provide energy to the host through short-chain fatty acids (SCFAs) [161,162,163]. They have also shown that free FA receptors expressed in enteroendocrine cells are involved in adipose tissue storage and hormonal balance and influence the timing of puberty [164,165].

To detect the mechanisms by which the gut microbiota triggers PP, Bo et al. created a PP model by feeding female mice with an HFD from 21 days old [19]. After puberty, the serum hormone levels, gut microbiome sequencing, and metabolomics were collected [19]. The authors found that an HFD after weaning led to PP and increased serum estradiol, leptin, deoxycholic acid, and GnRH in the hypothalamus [19]. Moreover, through correlation analysis, GnRH positively correlated with specific bacteria (*Desulfovibrio, Lachnoclostridium, GCA-900066575, Streptococcus, Anaerotruncus*, and *Bifidobacterium*), suggesting that these bacteria may promote pubertal development [19]. In the same study, the researchers demonstrated that transplanting so-called “HFD-microbiota” promoted the PP of mice [19]. According to these results, an HFD’s effect on PP is regulated by the interaction of gut microbiota and hormones [19].

#### 5.2.4. Overexpression of p53 through Lin28/let-7 System

The transcriptional factor p53 is a central hub of gene networks in controlling pubertal onset [166]. This transcriptional factor also plays a crucial role in metabolism [166]. In light of these assumptions, Chen et al. examined the expression levels of p53 and Lin28/let-7 axis components in the hypothalami of HFD-fed mice [21]. The authors observed the vaginal opening (VO), a marker of puberty, in normal-fed (controls) and HFD-fed mice and evaluated both the hypothalamus-specific expression levels of p53 and possible underlying mechanisms via the Lin28/let-7 axis [21]. Specifically, the researchers showed that HFD advances VO in rodents and that HFD-fed mice had higher p53 expression levels in the hypothalami than controls [21]. Importantly, in HFD-fed mice, the hypothalamus-specific overexpression of p53 induced earlier VO, whereas inhibiting p53 expression delayed VO [21]. These results suggest that p53 is an important regulator of metabolic control in puberty onset. In addition, the Lin28/let-7 axis is seen as a part of the gene network that controls puberty onset, as its potential impact on pubertal regulation was already revealed by a series of GWAS studies [167,168]. Chen et al., in the same study, found higher expression levels of c-Myc and Lin28b and lower let-7a levels in the hypothalami of HFD mice [21]. The authors proposed that the elevation of c-Myc and Lin28b levels could be a self-protective measure to compensate for the negative effects of HFD on glucose metabolism [166]. Moreover, overexpression of p53 inhibited c-Myc and Lin28b levels, whereas inhibition of p53 elevated c-Myc and Lin28b hypothalamic levels in HFD-fed mice [21]. These findings further suggest that the role of p53 in pubertal control is partly mediated by the Lin28/let-7 axis [21].

## 6. Conclusions

After weaning, an HFD can cause PP. Although evidence is scarce and studies limited, especially in the pediatric field, the harm of HFDs on PP is a relevant problem that cannot be ignored. These first pieces of evidence underline the important implications of HFDs for puberty; thus, further human studies would be useful and necessary to address the issue, in particular in boys. In fact, despite the fact that in male subjects there are limited studies on the association between early sexual maturation, obesity, and HFD, the link seems to be less evident than in females. This observation could be explained by a frequent negative association between early sexual maturation and obesity in males [132,169] and by a higher sensitivity to the central regulatory effects of some metabolic signals in females compared to males [170,171]. However, further scientific evidence is necessary to clarify.

Promoting HFD-avoiding behavior may be useful in preserving children’s physiological development and protecting reproductive health. Controlling HFDs may represent a target for policy action to improve global health.

## Figures and Tables

**Table 1:** Manuscripts analyzed to discuss the epidemiological evidence on the relation between HF content, obesity, and pubertal timing.

Authors	Journal/Year	Study Design	Population Involved (Sample Size and Age)	Main Results
**Sandhu J, et al. [15]**	*Int. J. Obes. (Lond).* ***2006***	A retrospective school-based cohort follow-up study	1520 men born between 1927 and 1956 with serial height and weight measurements from the age of 9 to 18, followed up in adulthood at a mean age of 63 years	Boys with a later puberty tended to be taller and less adipose as adults. Boys with a higher childhood BMI tended to have an earlier puberty.
**Euling SY, et al. [17]**	*Pediatrics.* ***2008***	Comparative study	Examination of US puberty timing data from 1940 to 1994	Trend toward an earlier breast development onset and menarche in girls, even if a minority of panelists concluded that the current data on girls puberty timing for any marker are insufficient. Current data for boys are, instead, insufficient to evaluate secular trends in male pubertal development.
**Chen C, et al. [132]**	*Erratum in: BMJ Open.* ***2017***	Population-based cross-sectional study	17,620 Chinese children aged 6–12 years.	25.98% and 38.58% of boys with precocious puberty were, respectively, accompanied by obesity or central obesity. 13.86% and 29.42% of girls with precocious puberty were, respectively, accompanied by obesity or central obesity.
**Rosenfield RL, et al. [135]**	*Pediatrics.* ***2009***	Population-based study	Comparison of stage 2 breasts, stage 3 (sexual) pubic hair, and menarche in the Third National Health and Nutrition Examination Survey sample of children with normal BMI with those with excessive BMI	Girls with excessive BMI had a significantly higher prevalence of breast appearance from ages 8.0 through 9.6 years and pubarche from ages 8.0 through 10.2 years than those with normal BMI.
**Szamreta EA, et al. [137]**	*Public. Health Nutr.* ***2020***	Population-based study	202 girls aged 9 or 10 years at baseline (2006–2014)	High Mediterranean-like diet adherence was associated with a later onset of thelarche and menarche.
**Koo MM, et al. [139]**	*Public Health Nutr.* ***2002***	Prospective cohort study	637 pre-menarcheal girls, 6 to 14 years of age	A higher intake of dietary fiber was associated with a later age at menarche.
**Berkey CS, et al. [140]**	*Am J Epidemiol. **2000***	Longitudinal study	67 Caucasian girls, birth to 10 years of age	Girls who consumed more animal protein and less vegetable protein at ages 3–5 years had earlier menarche Girls aged 1–2 years with higher dietary fat intakes and girls aged 6–8 years with higher animal protein intakes became adolescents with earlier peak growth.
**Mervish NA, et al. [141]**	*Nutr Res.* ***2013***	Longitudinal study	1178 girls, aged 6–8 years	Highest flavonol consumption was associated with later breast development (even if there are different limitations of the study).
**Jansen EC, et al. [143]**	*J Nutr.* ***2015***	Prospective study	456 girls aged 8.4 ± 1.7 years, followed for a median 5.6 years	Higher red meat intake is associated with an earlier age at menarche. Higher fatty fish intake is associated with a later menarcheal age.
**Mueller NT, et al. [144]**	*Am J Clin Nutr.* ***2015***	Prospective study	2379 girls (1213 African American, 1166 Caucasian) aged 9–10 years, followed for 10 years	Consumption of caffeinated and artificially sweetened soft drinks was positively associated with risk of early menarche.
**Günther AL, et al. [145]**	*J Nutr.* ***2010***	Data collected from the longitudinal Dortmund Nutritional and Anthropometric Longitudinally Designed Study	112 participants between 6 and 13 years	A higher total animal protein intake at 5–6 y was related to an earlier pubertal growth spurt. A higher vegetable protein intake at 3–4 and 5–6 y was related to later pubertal growth spurt.
**Hoppe C, et al. [146]**	*Am J Clin Nutr. **2004***	Data analysis	90 children (54 boys), aged 2.5 years	Milk intake was positively associated with a stimulating effect on sIGF-I concentrations and, thereby, on growth.
**Wiley AS [147]**	*PLoS ONE.* ***2011***	Regression analysis from data collected in NHANES 1999–2004	2657 women age 20–49 years; 1008 girls age 9–12 years	Greater milk intake is associated with an increased risk of early menarche or a lower age at menarche.
**Carwile JL, et al. [150]**	*J Nutr.* ***2015***	Prospective study	5583 US girls aged 9–14 years	Frequency of milk consumption seems not to be correlated with the onset of menarche.
**Merzenich H, et al. [149]**	*Am J Epidemiol. **1993***	Prospective study	261 girls, aged 8–15 years	Increased fat intake was associated with accelerated menarche. Increased sports activity was associated with a delay in menarche.
**Rogers IS, et al. [148]**	*Public Health Nutr.* ***2010***	Avon Longitudinal Study of Parents and Children	3298 girls aged 3–10 years	Higher intakes of protein and meat in early to mid-childhood may lead to earlier menarche.

## Data Availability

Not applicable.
